# Editorial: Role of Epigenetics in Autoimmune Diseases

**DOI:** 10.3389/fimmu.2020.01284

**Published:** 2020-06-19

**Authors:** Marzia Dolcino, Simonetta Friso, Carlo Selmi, Claudio Lunardi

**Affiliations:** ^1^Department of Medicine, University of Verona, Verona, Italy; ^2^Rheumatology and Clinical Immunology, Humanitas Clinical and Research Center, IRCCS, Milan, Italy; ^3^Department of Biomedical Sciences, Humanitas University, Milan, Italy

**Keywords:** epigenetic, autoimmune diseases, DNA methylation, micro RNA, biomarkers

Epigenetics is the study of gene modifications that are heritable but not caused by changes in the DNA sequence, ultimately modulating gene expression and thus regulating cell functions. Epigenetic features refer to DNA methylation and hydroxymethylation, histone modifications, and non-coding RNAs, which all contribute to play a role in the development of autoimmunity.

In the last few years several studies have been performed to highlight the contribution of epigenetic mechanisms to the pathogenesis of autoimmune diseases. In the present Research Topic we collected some papers that dissected the role played by epigenetics in autoimmune diseases and identified some epigenetic factors that may serve as biomarkers for disease status, prognosis, and response to treatment. One major and intuitive example of the role of epigenetics in the determination of autoimmune diseases is based on the study of monozygotic twins discordant for specific conditions. Quite surprisingly, the vast majority of autoimmune disorders share concordance rates which are well below 50% in genetically identical twins, thus making the genomic predisposition likely necessary but insufficient for disease onset (1). Of note, monozygotic twins increase the epigenetic differences with age and this may well-represent a model by which twins become clinically discordant (2). In general terms, monozygotic twins have been recently used to investigate the hereditary component of the immune function and data have clearly demonstrated that the majority of our immune readouts are largely independent of the genomic predisposition (3). In support of this notion, monozygotic twins discordant for being or not affected by systemic lupus erythematosus, have been observed to show significant differences in both global and gene specific DNA methylation that paralleled with expression modifications of relevant genes for the disease, thus further highlighting the critical role of epigenetics in autoimmune diseases (4). Recently, also Long non coding RNAs have been linked to the pathogenesis of autoimmune diseases by modulating genes expression both directly and indirectly, through targeting microRNAs (miRNAs) (5–7).

The review by Wu et al. represents a comprehensive analysis of the state of studies on different epigenetic deregulations including abnormal DNA methylation, histone modifications, aberrant non-coding RNAs that can be important in several autoimmune diseases such as systemic lupus erythematosus (SLE), rheumatoid arthritis (RA), psoriasis, type 1 diabetes (T1D), and systemic sclerosis (SSc).

The Authors also discuss the possibility of utilization of specific epigenetic modifications as potential biomarkers and novel therapeutic targets for these diseases.

The review by Surace and Hedrich analyses all the above mentioned epigenetic deregulations not only in classical autoimmune diseases but also in the autoinflammatory disorders.

A study by Andonian et al. aimed at identifying the relationships between six RA-related miRNAs and RA disease activity, inflammation, metabolic parameters and metabolic function.

The six miRNAs studied correlate with either inflammatory markers, adiposity and cardiometabolic risk markers. The paper indicates that such miRNAs may represent an epigenetic link between RA inflammation and cardiometabolic comorbidities.

Santos et al. demonstrate an increased level of miR-101-3p in Type 1 Diabetes (T1D) patients and in subjects with normal glucose levels but positive for multiple autoantibodies, thus suggesting that altered levels of this miRNA may predict the onset of the disease.

Differentially expressed non-coding circular RNAs (circRNAs) are thought to play a role in the progress of autoimmune diseases. The paper by Guo et al. analyses the role of circRNAs in SLE. In this paper the Authors identify a circRNA (Hsa_circ_0000479) that may represent a promising disease marker in SLE.

Genome-wide DNA methylation studies demonstrated significant methylation changes in different autoimmune diseases. In this regard Carnero-Montoro et al. used this approach in Mixed Connective Tissue Disease (MCTD) showing hypomethylation in several genes involved in immune-related functions. These epigenetic modifications are typical of MCTD and cannot be found in patients with SLE and SSc.

Another study by Imgenberg-Kreuz et al. performed a cross-comparative analysis of DNA methylation in patients with SLE and in patients with primary Sjögren's syndrome (pSS). The Authors show that the majority of differential DNA methylation is shared between the two diseases with some important differences. The study also highlights neutrophil dysregulation as a shared mechanism, thus emphasizing the role of neutrophils in the pathogenesis of systemic autoimmune diseases.

Finally, a paper by Karouzakis et al. performed the first epigenomic characterization of lymph node stromal cells (LNSCs) during health and early RA, by analyzing their transcriptome and DNA methylome.

Interestingly, DNA methylation analyses show common and specific differentially methylated CpG sites in LNSC from RA patients compared with healthy subjects.

Notably these CpG sites are all associated with immune functions such as antigen processing and presentation.

The topic of this issue highlights the important role of research in the field of epigenetics in order to identify new disease biomarkers and therapeutic targets in autoimmune diseases.

## Author Contributions

All authors listed have made a substantial, direct and intellectual contribution to the work, and approved it for publication.

## Conflict of Interest

The authors declare that the research was conducted in the absence of any commercial or financial relationships that could be construed as a potential conflict of interest.

## References

[1] Generali E Ceribelli A Stazi MA Selmi C. Lessons learned from twins in autoimmune and chronic inflammatory diseases. J Autoimmun. (2017) 83:51–61. 10.1016/j.jaut.2017.04.005 28431796

[2] Fraga MF Ballestar E Paz MF Ropero S Setien F Ballestar ML., Epigenetic differences arise during the lifetime of monozygotic twins. Proc Natl Acad Sci USA. (2005) 102:10604–9. 10.1073/pnas.0500398102 16009939 PMC1174919

[3] Brodin P Jojic V Gao T Bhattacharya S Angel CJ Furman D. Variation in the human immune system is largely driven by non-heritable influences. Cell. (2015) 160:37–47. 10.1016/j.cell.2014.12.020 25594173 PMC4302727

[4] Javierre BM Fernandez AF Richter J Al-Shahrour F Martin-Subero JI Rodriguez-Ubreva J. E. Changes in the pattern of DNA methylation associate with twin discordance in systemic lupus erythematosus. Genome Res. (2010) 20:170–9. 10.1101/gr.100289.109 20028698 PMC2813473

[5] Dolcino M Tinazzi E Vitali C Del Papa N Puccetti A Lunardi C. Long non-coding RNAs modulate sjögren's syndrome associated gene expression and are involved in the pathogenesis of the disease. J Clin Med. (2019) 8:1349. 10.3390/jcm8091349 31480511 PMC6780488

[6] Dolcino M Tinazzi E Puccetti A Lunardi C. Long non-coding RNAs target pathogenetically relevant genes and pathways in rheumatoid arthritis. Cells. (2019) 8:816. 10.3390/cells8080816 31382516 PMC6721587

[7] Dolcino M Tinazzi E Puccetti A Lunardi C. In systemic sclerosis, a unique long non coding rna regulates genes and pathways involved in the three main features of the disease (vasculopathy, fibrosis and autoimmunity) and in carcinogenesis. J Clin Med. (2019) 8:320. 10.3390/jcm8030320 30866419 PMC6462909

